# Medical marijuana for the treatment of vismodegib-related muscle spasm

**DOI:** 10.1016/j.jdcr.2017.06.012

**Published:** 2017-09-01

**Authors:** Joyce T. Yuan, Tiffany L. Tello, Carter Hultman, Christopher A. Barker, Sarah T. Arron, Sue S. Yom

**Affiliations:** a Department of Dermatology, University of California, San Francisco, San Francisco, California; b Department of Radiation Oncology, University of California, San Francisco, San Francisco, California; c Department of Radiation Oncology, Memorial Sloan Kettering Cancer Center, New York, New York

**Keywords:** basal cell carcinoma, medical marijuana, muscle cramps, vismodegib, BCC, basal cell carcinoma, CBD, cannabidiol, FDA, US Food and Drug Administration, MM, medical marijuana, mS, multiple sclerosis, THC, tetrahydrocannabinol

## Introduction

Basal cell carcinoma (BCC) arises from loss-of-function mutations in tumor suppressor patched homologue 1, which normally inhibits smoothened homologue in the sonic hedgehog signaling pathway.^1^, ^2^ Vismodegib, a smoothened homologue inhibitor,^2^ is US Food and Drug Administration (FDA) approved for metastatic or locally advanced BCC that has recurred after surgery or for patients who are not candidates for surgery and radiation.^1^, ^2^ Common adverse effects of vismodegib are muscle spasms, alopecia, dysgeusia, nausea, and weight loss.^1^, ^2^ Muscle spasms worsen with duration of drug administration and may lead to drug discontinuation.^1^, ^2^ We report a case of vismodegib-related muscle spasm that was successfully treated with medical marijuana (MM).

## Case report

A 58-year-old woman presented with a 10-year history of a painful and bleeding skin lesion. Examination found an 8- × 13-cm ulcerated and indurated plaque with rolled borders covering the forehead and both upper eyelids. Biopsy confirmed nodular BCC. Computed tomography and magnetic resonance imaging found no perineural invasion or intracranial extension. Surgery was deemed exceptionally morbid, and she was offered a phase II clinical trial (NCT01835626), consisting of 14 weeks of induction vismodegib (150 mg/d) followed by 7 weeks of vismodegib concurrent with radiation (70 Gy in 35 fractions). Within the first 3 weeks of vismodegib, the patient had muscle spasms in her calves (grade 2), nausea (grade 2), and dysgeusia (grade 1). Nausea resolved with ondansetron. Baclofen failed to improve spasms and caused sleepwalking, leading the patient to refuse the medication. A month later, she reported persistent grade 2 muscle spasms, now including her hands and feet. Cyclobenzaprine produced no effect. During the first week of vismodegib and radiation, the patient started MM, having heard of its indication in the treatment of muscle cramps. She smoked 3 to 4 joints daily of Trainwreck strain, containing 18.6% tetrahydrocannabinol (THC), 0.0% cannabidiol (CBD), and 0.0% cannabinol. Her muscle spasms resolved immediately. She continued MM for 3.5 weeks, until the cost of MM became prohibitive. She reported no adverse effects from MM. Complete resolution of muscle spasms was sustained through the remaining 3.5 weeks of vismodegib. Complete blood count, comprehensive metabolic panel, and lactate dehydrogenase level were monitored throughout the study with no significant changes. As of 18 months posttreatment, the patient had a complete clinical response of her BCC.

## Discussion

At the time of this writing, MM is legal in 28 states, Washington DC, Guam, and Puerto Rico^3^; more widespread legalization is likely.^3^, ^4^ Marijuana contains more than 400 compounds including terpenoids, flavonoids, and approximately 70 different cannabinoids. Many cannabinoids have been studied for medicinal purposes, including THC and CBD.^5^ Dronabinol (Marinol, AbbVie, North Chicago, IL and Syndros, Insys Therapeutics, Chandler, AZ), a synthetic oral form of THC, is FDA approved for anorexia and weight loss associated with HIV and for chemotherapy-related nausea and vomiting. Nabilone (Cesamet, Valeant Pharmaceuticals, Laval, Quebec, Canada), another synthetic oral form of THC, is FDA approved for refractory chemotherapy-induced nausea and vomiting.^6^ Other cannabinoid compounds have been studied for chronic and postoperative pain, fibromyalgia, asthma, glaucoma, epilepsy, and musculoskeletal conditions.^6^, ^7^ Epidiolex (GW Pharmaceuticals, Cambridge, United Kingdom), a purified CBD compound, reduced convulsive seizures related to Dravet syndrome, a severe form of childhood epilepsy, in a recent phase III trial.^8^ Sativex (GW Pharmaceuticals, Cambridge, United Kingdom), a formulated extract of *Cannabis sativa*, contains both THC and CBD and is licensed in 16 countries outside the United States for treatment of multiple sclerosis (MS)-related spasticity.^9^

Management of muscle spasms with cannabinoids has been studied most in patients with MS and spinal cord injury.^4^, ^6^, ^7^, ^10^ In a pooled analysis of 3 clinical trials, nabilone and nabiximols modestly improved muscle spasticity compared with placebo, as reported by MS patients; an average improvement of 0.76 on a 0-to-10 numerical rating scale was observed.^6^, ^10^ Clinician-measured spasticity using the Ashworth scale, which grades muscle tone in response to passive motion, trended toward improvement in a pooled analysis of 5 trials investigating the benefit of nabiximols, dronabinol, and THC/CBD^6^ in MS patients.^6^ Overall, by meta-analysis, moderate-quality data support the use of cannabinoids for spasticity and chronic pain in MS patients.^6^, ^11^ For muscle spasm from spinal cord injury, the limited evidence available suggests benefit from cannabinoids.^9^ In one pilot study, use of nabilone resulted in significant improvement by Ashworth scale and a trend toward improvement on other spasticity scales.^12^ Persistent spasms are an approved prescribing indication for MM in California. In a study of 1,746 patients from 9 California clinics, pain and muscle spasm were the most common reasons for prescribing.^13^

The mechanism of cannabinoid-associated antispasmodic activity is not well understood. Activity at CB1 and CB2 receptors, located in high concentrations at the cerebellum and substantia nigra, may play a role.^7^, ^14^ Other hypotheses include decreased perception of muscle spasm, euphoria, and placebo effect.^7^ Nabiximols, dronabinol, nabilone, THC, and ECP002A are cannabinoid subtypes that have been investigated for relief of muscle spasticity; any difference in efficacy based on cannabinoid subtype is not established.^6^

Muscle spasm is a frequent side effect of vismodegib.^1^, ^2^ In one phase II study, 68% of patients on vismodegib experienced muscle spasms; 48% were grade 1, 16% grade 2, and 4% were grade 3 or 4 in severity.^2^ Similar frequencies are seen in other studies.^1^ There is no standard treatment for this side effect. Antispasmodics such as cyclobenzaprine, baclofen, and quinine are commonly tried.^15^ Calcium channel blockade with amlodipine has some efficacy,^16^ and a clinical trial evaluating the effect of levocarnitine is ongoing (NCT01893892). Use of cannabinoids has not been reported.

In the phase III Epidiolex study, the most common adverse events were somnolence, diarrhea, decreased appetite, fatigue, pyrexia, vomiting, lethargy, upper respiratory tract infection, and convulsion, with 84% reporting their symptoms to be mild or moderate.^8^ MM may also exacerbate substance abuse or pre-existing psychiatric illness,^4^, ^7^ with anecdotal reports of psychosis, but the incidence and exact relation to marijuana are not well studied.^7^ Our patient had a long-standing history of anxiety and depression for which she took venlafaxine and aripiprazole; she did not experience worsening of her psychiatric disease from MM.

One marijuana joint contains, on average, 0.66 g of marijuana,^17^ although the definition of a joint is highly variable. With any MM formulation, patients should start at a low dose and gradually titrate to effect.^18^ Additional studies could confirm safety and efficacy and better specify the optimal cannabinoid subtypes, preparations, and dosages that may be most beneficial for vismodegib-induced muscle spasms.
